# Gastrointestinal autonomic nerve tumours – report of a case and review of literature

**DOI:** 10.1186/1477-7819-3-46

**Published:** 2005-07-19

**Authors:** Manoj H Mulchandani, Dipankar Chattopadhyay, John O Obafunwa, Vickram B Joypaul

**Affiliations:** 1 Department of Surgery, South Tyneside District Hospital, Harton Lane, South Shields, Tyne and Wear, NE34 OPL, UK; 2 Department of Pathology, South Tyneside District Hospital, Harton Lane, South Shields, Tyne and Wear, NE34 OPL, UK

## Abstract

**Background:**

Gastrointestinal autonomic nerve tumours are uncommon stromal tumours of the intestinal tract. They can involve any part of the gastrointestinal system, but are very rarely seen in the rectum.

**Case presentation:**

We report a unique case of rectal schwannoma with associated synchronous adenocarcinoma of the splenic flexure and adenoma of the descending colon. A 70-year-old patient was admitted with complaint of bleeding per rectum and investigations revealed the presence of a large submucosal rectal lesion in addition to the colonic pathologies. Following panproctocolectomy with permanent spout ileostomy, histopathology and immunohistochemistry confirmed the rectal lesion to be a schwannoma.

**Conclusion:**

Literature review of the few reported cases has suggested radical surgical excision to be the best approach. Prognosis tends to be favourable after resection.

## Background

The gastrointestinal autonomic nerve tumours (GANTs) were first described and defined by Herrera *et al*, in 1984 [1]. GANTs are uncommon stromal tumours accounting for 0.1% of benign tumours of the gastrointestinal tract [2]. It is a subgroup of gastrointestinal stromal tumours (GISTs) with specific ultrastructural differences; suggesting its origin from the myenteric plexus [3]. Schwannomas belong to this group and may develop practically in any anatomic region [2].

Without immunohistochemical studies, Schwannomas are often misdiagnosed as leiomyomas or leiomyosarcomas and generally present as an asymptomatic mass and/or with non-specific symptoms of fatigue and prolonged pain as well as signs of low grade pyrexia, anaemia and haemorrhage. Conventional pathological techniques are usually not diagnostic [2]; electron microscopy often being required to establish the diagnosis of GANTs and to exclude them from other gastrointestinal tumours [4].

Common sites for GANTs include the stomach, duodenum, jejunum, ileum [5] and to date literature search has revealed only twenty cases of colonic schwannomas and only four reported cases of rectal schwannomas [2,6-9]. We report a rare case of rectal schwannoma who also had incidental adenocarcinoma and adenoma of the colon.

## Case presentation

A 70-year-old gentleman presented with a two-month history of bleeding per rectum and altered bowel habits. There was no history of tenesmus but sensation of incomplete evacuation following defecation as well as a feeling of a lump in the anal canal was present. His appetite and weight had been stable.

Clinically he looked well and abdominal examination revealed no abnormality. Per rectal examination revealed a mass in the posterior wall of the rectum with intact mucosa, which had significantly narrowed the lumen. Colonoscopy revealed the presence of a mass in the posterolateral wall of the rectum extending from 4 to 14 cm from the anal verge, with normal overlying mucosa. In addition, there was also a large sessile polyp in the descending colon and an ulceroproliferative growth at the splenic flexure; histopathology confirmed adenomatous polyp and adenocarcinoma respectively.

**Figure 1:**
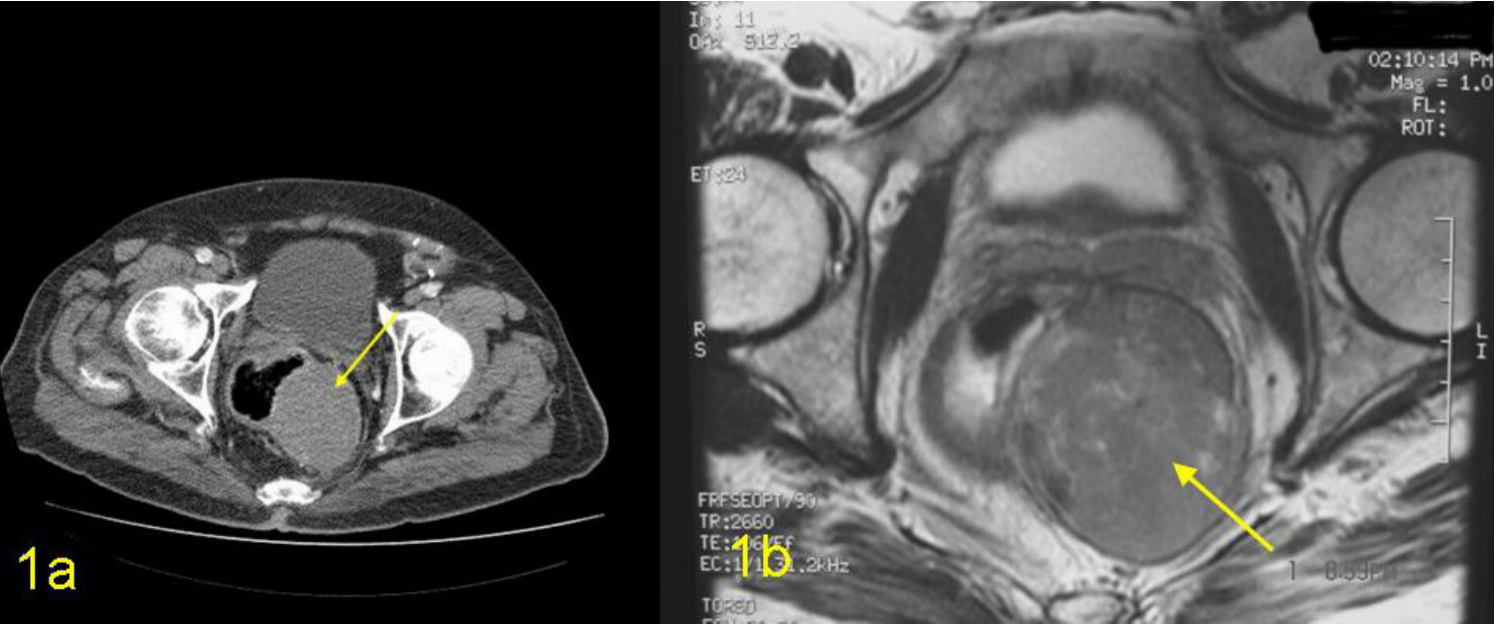
Imaging of the large bowel 1a) CT scan showing the large lesion in the posterior and left lateral wall of the rectum and 1b) MRI imaging: the distal rectal wall lesion (GANT) is shown to be intramural and free from adjoining structures.

**Figure 2:**
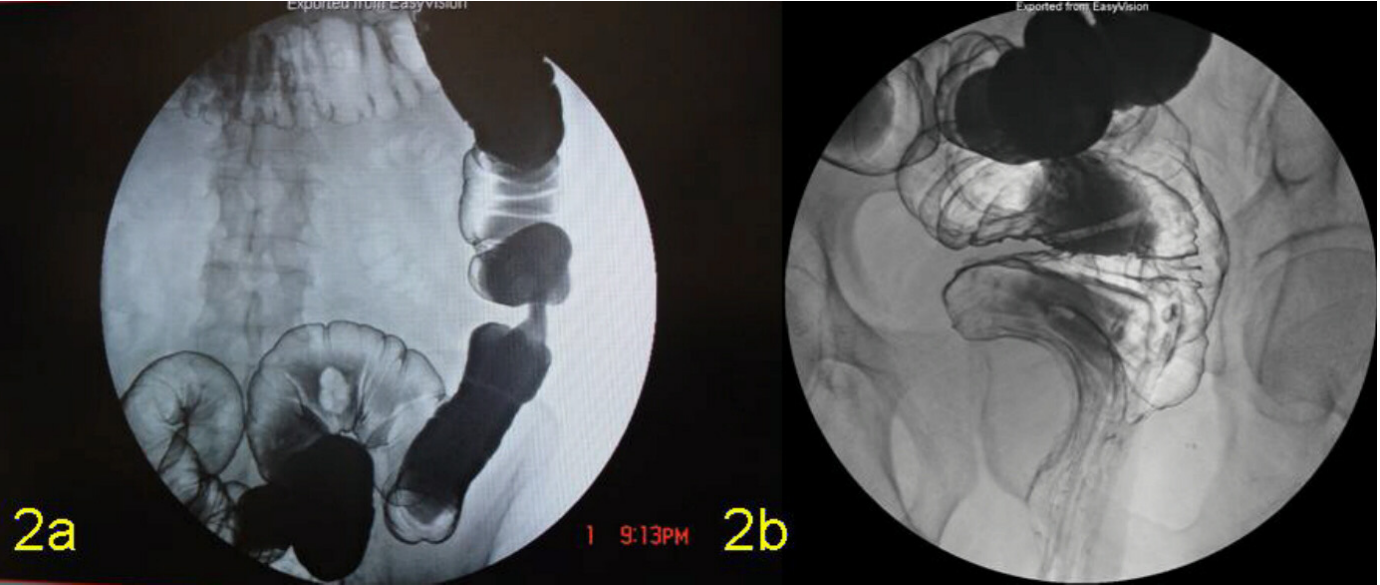
Barium enema **a) **the apple core lesion at the splenic flexure and **b) **the extrinsic compression in the distal rectum caused by schwannoma.

Computerised tomography (CT) of the pelvis showed thickening of the distal rectum with an obvious low density poorly enhancing lesion with possible calcification in its wall. Tissue plane between this lesion and prostate was not clearly seen but there was no infiltration of the ischiorectal fat (Figure 1a). Magnetic resonance imaging (MRI) showed a very large well-defined mass in the left presacral / ischio-anal fossa contiguous with rectal wall and thus almost certainly arising from one of the elements of the rectal wall (Figure 1b). Barium enema showed an apple core lesion in the splenic flexure (Figure 2a) as well as narrowing of the distal rectal lumen (Figure 2b).

The case was fully discussed at the colorectal multidisciplinary team meeting and all the surgical options including left hemicolectomy combined with rectosigmoidectomy plus transverse colostomy were fully evaluated. However, the consensus was that the additional presence of an adenocarcinoma as well as an adenoma higher up in the colon combined with the size of rectal schwannoma (12 cms) made panproctocolectomy with permanent ileostomy the best viable option. Furthermore, this was also the preferred choice of the patient as he opted to have an ileostomy rather than a colostomy. Surgery was performed without complications and the postoperative recovery was uneventful. At 6 months follow-up, the patient remains asymptomatic.

**Figure 3:**
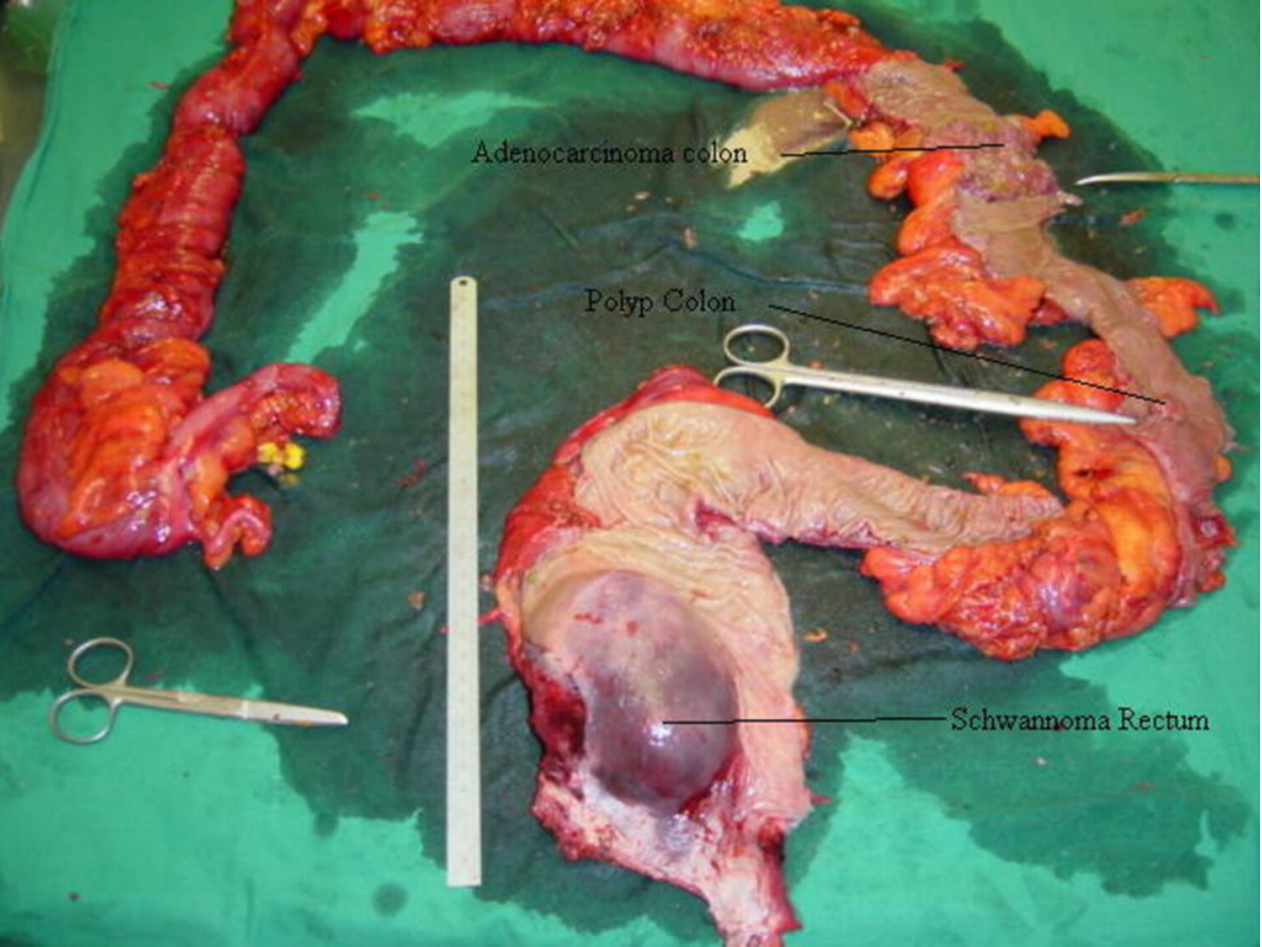
Resected specimen showing malignant tumour at the splenic flexure, polyp in the descending colon and rectal schwannoma.

Macroscopic examination of the resected specimen confirmed the malignant tumour in the splenic flexure, the polyp in the descending colon and a 10 × 12 cm tumour with a well-defined pseudocapsule in the postero-lateral wall of the mid and low rectum (Figure 3). The surface of the latter lesion was fusiform with bluish-grey colour and its cut surface was irregularly lobulated with occasional cystic and necrotic areas.

**Figure 4:**
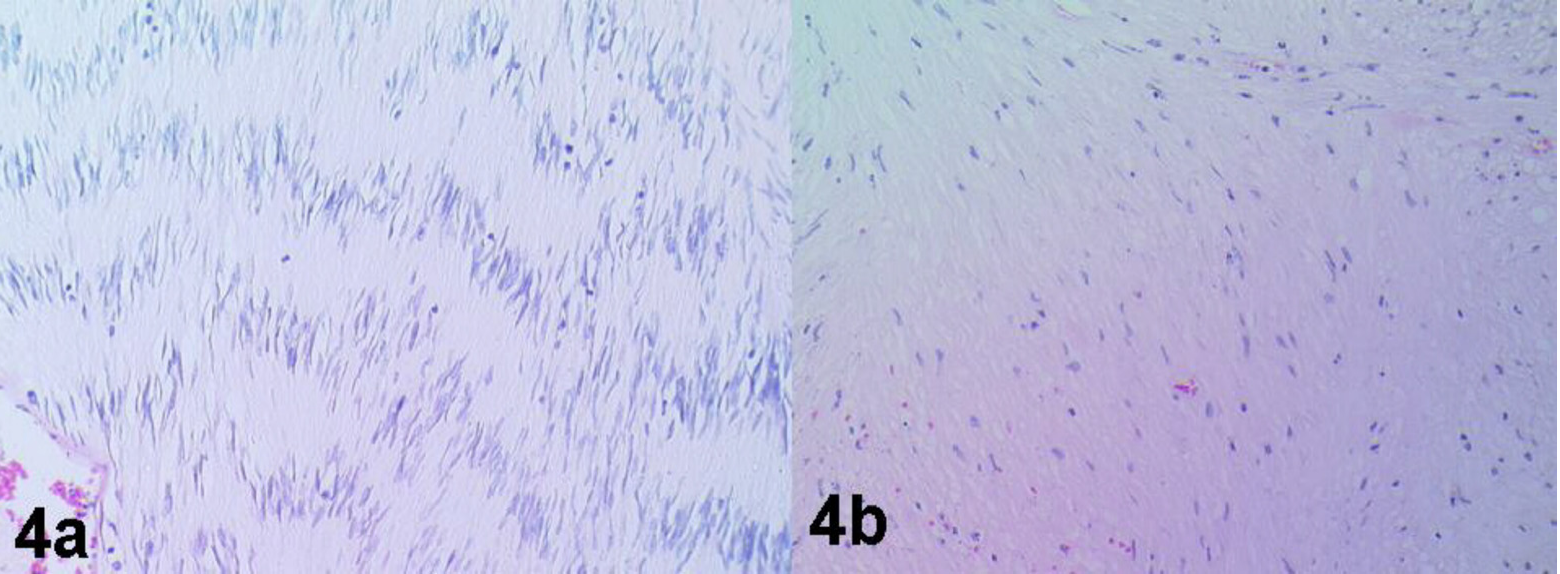
Photomicrograph showing a) cellular (Antoni A) and b) myxoid (Antoni B) areas (hematoxylin and eosin ×40).

**Figure 5:**
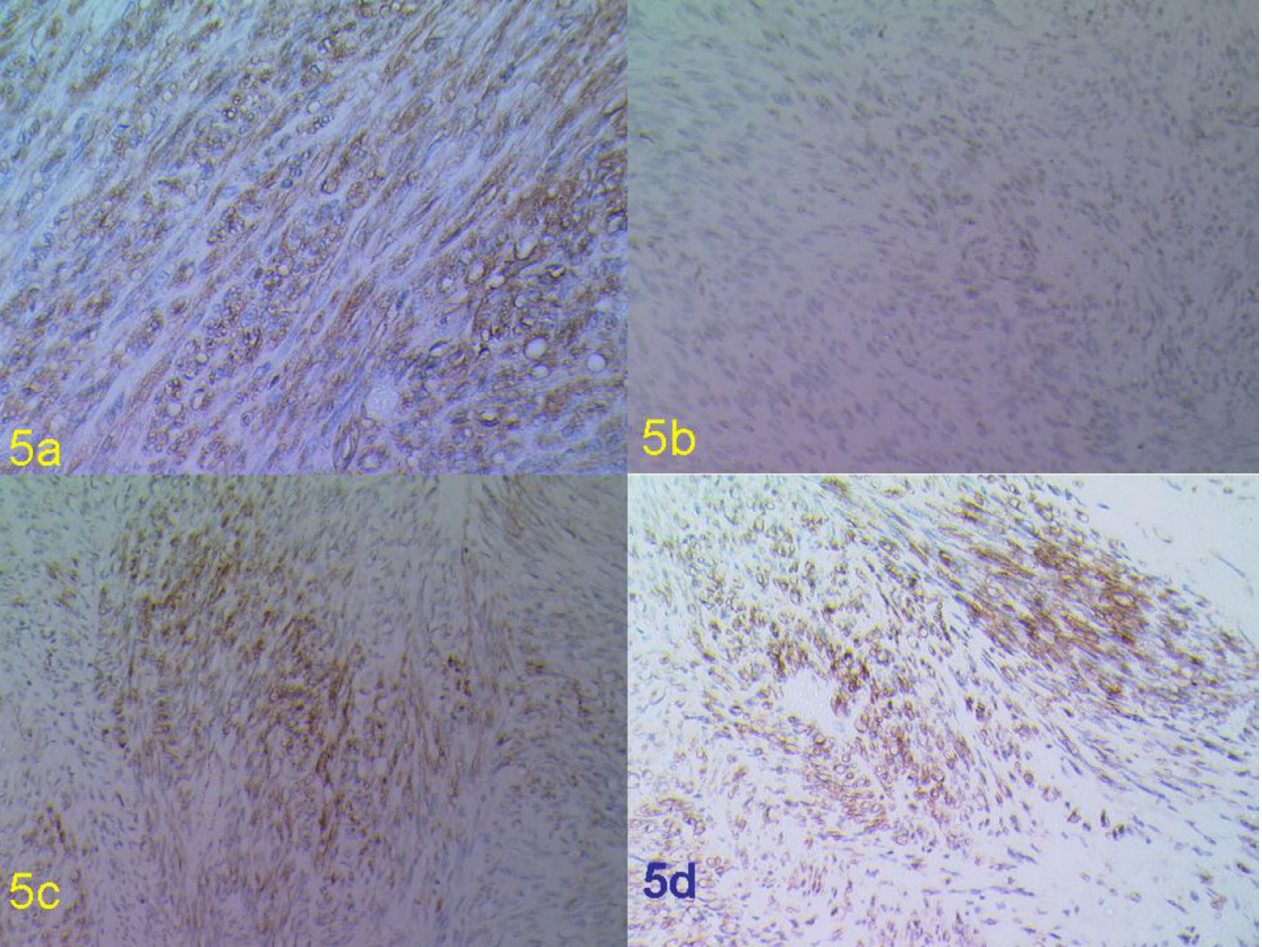
Immunohistochemical staining of the rectal lesion with a) CD34, b) S100, c) Vimentin and d) NSE (original magnification ×40).

Histopathology of the splenic flexure lesion showed an invasive moderately differentiated adenocarcinoma (G1; Dukes B; Stage2). The polyp further down the colon was a tubulovillous adenoma with moderate focal dysplasia. The rectal tumour showed irregularly running fascicles of spindly to plump mesenchymal cells. The tumour was fairly vascularised with presence of a number of dilated and engorged vessels. The mitotic count was 4 per high power field. Some parts of the tumour were quite cellular while few areas were hypocellular reminiscent of the Antoni A and Antoni B patterns respectively (Figures 4a and 4b). Immunostaining revealed strong reactivity for neuron specific enolase (NSE), vimentin, CD34 and focal positivity for S100 (Figures 5a, 5b, 5c and 5d). Smooth muscle markers actin and desmin were negative.

## Discussion

Gastrointestinal autonomic nerve tumours represent a distinct but rare subcategory of gastrointestinal stromal tumours accounting for 1% of all malignant gastrointestinal tumours. Initially described as plexosarcomas, these tumours have been reported to be more common in males and have a wide spectrum of age range [5]. Literature review has shown rare association of GANTs with neurofibromatosis [10-12] and adrenal ganglioneuroma [13]. Schwannomas are types of gastrointestinal tract autonomic tumours and amongst this group, rectal schwannomas are very rare [13,14]. Furthermore, the case described here, was associated with adenocarcinoma and adenoma of the colon (Figure 3) and to date, there has been no reports of a similar association. Chance or an epigenetic event could explain such association of the different synchronous lesions.

Conventional imaging modalities such as barium enema, colonoscopy, computerised tomography and MRI have been used to investigate these patients but there are no definite radiological criteria to differentiate benign from malignant stromal tumour [15]. As shown in Figures 1a and 1b, CT and MRI scans located the large encapsulated lesion as arising from the rectal musculature, but yielded no additional information which allow for the distinction of benign from malignant potential. Recently Levy *et al*, [16] have reported that CT imaging may help to differentiate GANTs from gastrointestinal stromal tumours (GISTs). Low attenuation features (indicative of haemorrhage, necrosis and degeneration typically found in the centre of the GISTs) was not seen in our case (Figure 1a); thus favouring GANTs. Endoscopic ultrasound has been suggested to be reliable in predicting malignancy and the predictive features being irregular margins, depth of penetration, cystic spaces and lymph nodes with a malignant pattern [17]. However, it does not differentiate GANTs from the other stromal tumours [15]. Endoscopic ultrasound guided fine needle aspiration with immunohistochemical analysis may be useful in the preoperative diagnosis of GIST [18] but in our patient, this was not done, as it would not have altered the treatment, due to the presence of synchronous tumours in the colon and also the size of the lesion.

Although GANTs do exhibit a variety of specific histological features, Lauwers *et al*, [10] believe that neither individual cell characteristics nor the growth pattern of these tumours allow distinction from GISTs. The differentiation of schwannomas from other stromal tumours is important because the latter group has high-risk of malignant behaviour [19]. Furthermore, whilst some authors disagree [5], others have suggested the presence of both Antoni A / Verocay bodies (cells forming a typical palisade arrangement in a well-organised pattern) and Antoni B (small lacunar foci with loss of palisade architecture) areas to be very specific for schwannoma [20]. Such patterns have been clearly demonstrated in our case (Figures 4a and 4b).

Immunohistochemical studies of GANTs have usually demonstrated positivity to vimentin, CD34 and CD117 [5,10,21]. Positive reactivity with neuron specific enolase (NSE), S-100 protein, synaptophysin, and chromogranin A (proteins expressed by neurons from the autonomic nerve plexus) have also been reported and this support the histogenesis of GANTs from the autonomic plexus of Meissner or Auerbach [5]. Furthermore, positive immunoreactivity to the S-100 protein and Leu7 antigen tend to indicate the Schwannian nature of the tumour [22], whereas positivity to the glial fibrillary acidic protein point towards a myenteric plexus origin [3]. GANT is an ultrastructural variant of GIST, based on its consistent CD117 positivity and the presence of GIST-specific c-kit gene mutations in a significant number of cases [23]. The published GANT tumours have been variably and inconsistently S-100 protein positive. In our patient, immunohistochemistry revealed reactivity for vimentin, NSE, CD34 and for S-100 (Figure 5). Desmin and actin were negative.

Whilst immunohistochemistry provides important diagnostic differentiation of GANTs from other stromal tumours, definitive diagnosis can only be based on ultrastructural studies [5]. The ultrastructural criteria that suggest origin from myenteric plexus are neuron-like cells with long cytoplasmic processes containing microtubules, bulbous synapse like structures with dense core neurosecretory type granules and empty vesicles [10]. Ultrastructural examination is not available at most pathological units (including ours); this could explain the paucity of these cases and it is suggested that representative samples should be sent to centres having electron microscopic facilities.

The exact biological behaviour of GANTs is not yet fully elucidated due to the limited number of reported cases and as a result, determination of malignancy poses a difficult challenge, which cannot always be resolved by conventional histopathology. Various parameters have been studied in relation to tumour behaviour and to date; no single one is fully predictive of malignancy. Mitotic activity (counts >5 mitoses per high power fields) and tumour size (> 5 cm) tend to be associated with a high risk of metastasis or recurrence [24]. Although GANTs are generally considered benign, Lauwers *et al*, reported that 30% of these patients developed local recurrence [12] and as a result, radical surgery is the optimal treatment [5]. In our case, the additional presence of an adenocarcinoma and adenoma higher up in the colon combined with the size of the rectal schwannoma (>12 cms) made pan-proctocolectomy with permanent ileostomy the better option.

The recent finding of CD117 receptors in these tumours combined with technological advances has led to the development of anti-tumour agents. Treatment of such CD117 positive GANTs with tyrosine kinase inhibitors have been shown to be beneficial [25] and could in future, represent an appropriate form of palliative therapy in those patients with unresectable as well as metastatic tumours [5].

## Conclusion

Further studies are required not only to fully characterise the molecular biology of these tumours but also their aggressiveness as there is an inherent difficulty in making a benign diagnosis in GANTs. This is clearly important since it may result in a poor cross over in the assessment of the responsiveness of GANTs to anti-CD117 treatment.

## Competing interests

The author(s) declare that they have no competing interest.

## Authors' contributions

**MMH **collected the information, did literature search and wrote the manuscript.

**DC **assisted in writing the manuscript.

**OJO **assisted in pathological view and the microscopic pictures.

**JBV **helped in preparing the manuscript and edited the final version.

All authors have read and approved the final version.
